# Electrocardiographic imaging-aided stereotactic radioablation as an alternative therapy for refractory premature ventricular complexes

**DOI:** 10.1007/s10840-026-02266-1

**Published:** 2026-04-13

**Authors:** Marco Tomasino, Alba Santos-Ortega, Victoria M. Reyes, Raquel Granado, Alejandro Seoane-Ramallo, Ignacio Ferreira-González, Nuria Rivas-Gándara

**Affiliations:** 1 Medicine Department, Universitat Autónoma de Barcelona, Passeig Vall d’Hebron 119-131, Barcelona, 08023 Spain; 2 Cardiology Department, Vall d’Hebron University Hospital, Vall d’Hebron Research Institute (VHIR), Passeig Vall d’Hebron 119-131, Barcelona, 08023 Spain; 3 Department of Radiation Oncology, Vall d’Hebron University Hospital, Passeig Vall d’Hebron 119-131, Barcelona, 08023 Spain; 4 Medical Physics Department, Vall d’Hebron University Hospital, Passeig Vall d’Hebron 119-131, Barcelona, 08023 Spain; 5 Centro de Investigación Biomédica en Red de Enfermedades Cardiovaculares (CIBER-CV), Instituto de Salud Carlos III, Avenida Monforte de Lemos 3, Madrid, 28029 Spain

## Main text

A 78-year-old man with non-ischemic dilated cardiomyopathy and severe left ventricular systolic dysfunction was referred for treatment of frequent premature ventricular complexes (PVCs). His medical history included hypertension, type 2 diabetes mellitus, chronic obstructive pulmonary disease, and former tobacco use. A cardiac resynchronization therapy–defibrillator (CRT-D) had been implanted six years earlier for heart failure and left bundle branch block, and atrioventricular node ablation had subsequently been performed for refractory atrial tachycardia. Over the previous year, the patient developed frequent PVCs that reduced effective biventricular pacing and were associated with worsening heart failure symptoms (New York Heart Association class III) and deterioration of left ventricular ejection fraction to 25%.

**Figure:**
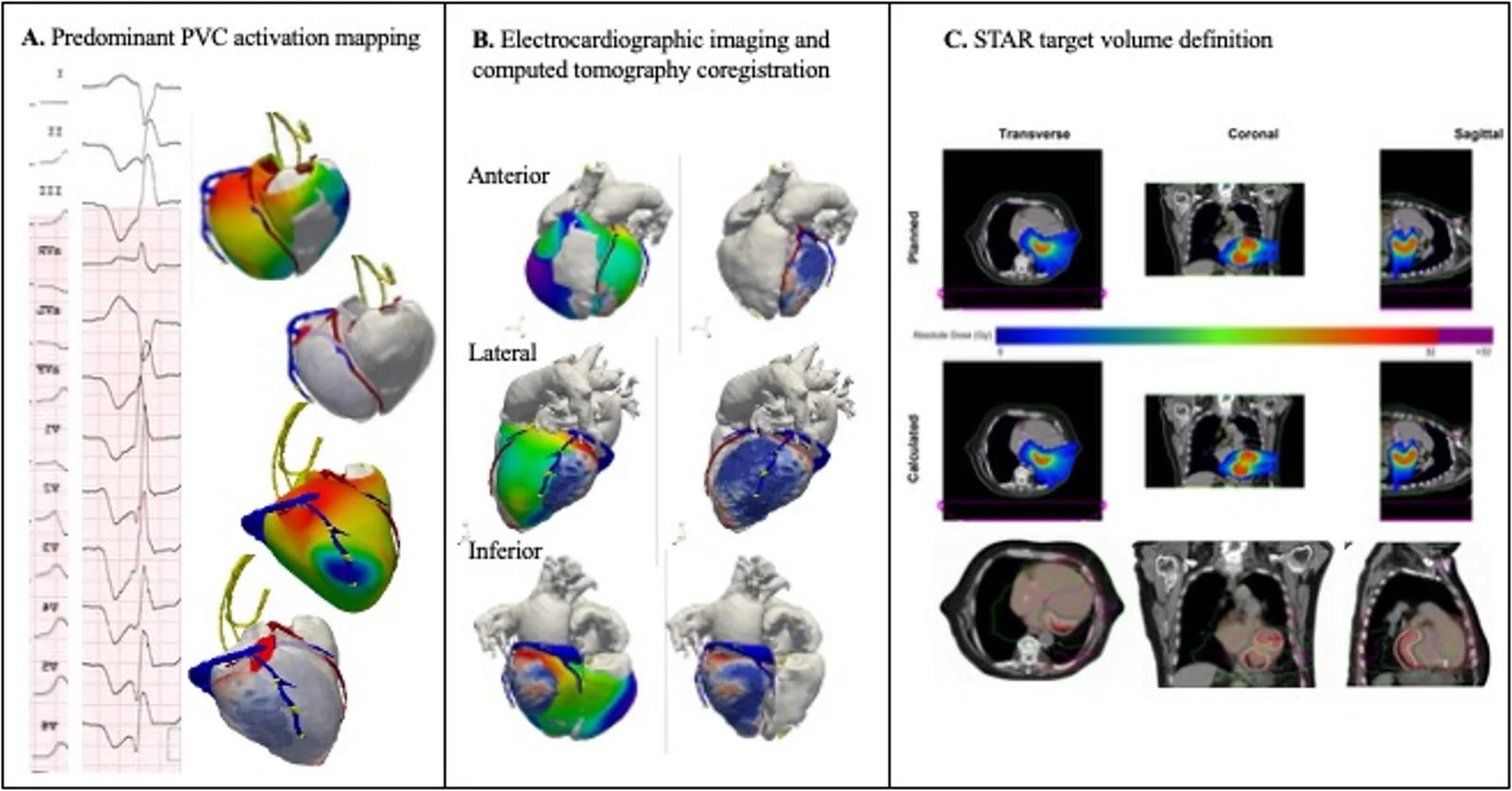

The predominant PVC showed a slurred onset, right bundle branch block morphology, positive precordial concordance, inferior axis, and negative aVL (Panel A). High-density electroanatomic mapping during a first ablation procedure localized the earliest activation to the laterobasal left ventricle; however, the earliest endocardial electrogram did not precede QRS onset, suggesting an epicardial origin. Endocardial radiofrequency ablation was ineffective. A second procedure aiming for epicardial access was attempted but aborted because of dense pericardial adhesions. Antiarrhythmic drug escalation, including mexiletine in addition to amiodarone and beta-blocker therapy, failed to reduce PVC burden and was poorly tolerated due to hypotension and asthenia.

At reevaluation, 24-hour Holter monitoring showed a PVC burden of 25% with a predominant morphology consistent with prior findings. Transthoracic echocardiography confirmed severe left ventricular dysfunction. Cardiac magnetic resonance imaging demonstrated dilated cardiomyopathy with myocardial fibrosis involving the basal and mid inferior and inferolateral segments, predominantly subendocardial. ECG-gated cardiac computed tomography revealed no obstructive coronary artery disease and allowed detailed anatomical assessment.

Given refractory symptoms, persistent high PVC burden impairing CRT function, and failure of both endocardial and epicardial catheter ablation, noninvasive stereotactic arrhythmia radioablation (STAR) was proposed [1]. For target definition, contrast-enhanced cardiac CT data were imported into the ADAS 3D software platform and integrated with noninvasive electrocardiographic imaging (ECGi) acquired using a 128-electrode body surface mapping system (Panel B). Analysis of myocardial wall thickness identified areas of thinning in the laterobasal left ventricle, colocalizing with fibrosis detected on cardiac magnetic resonance imaging.

Prolonged ECGi monitoring identified the clinically dominant PVC and two additional frequent PVC morphologies. Activation maps demonstrated that the earliest activation sites of all three PVC originated from adjacent regions along the lateral left ventricular annulus. This region corresponded precisely with the area of myocardial fibrosis and the proximal electrodes of the left ventricular CRT lead. Using this multimodal integration, a clinical target volume of 74.5 mL was delineated and fused with a four-dimensional planning CT scan (Panel C). STAR was delivered to the target volume with a single fraction of 25 Gy.

Twenty days after treatment, the patient presented with chest pain and peripheral oedema. Laboratory testing showed elevated inflammatory markers, and transthoracic echocardiography revealed a moderate pericardial effusion with bilateral pleural effusions. A diagnosis of inflammatory pleuropericarditis related to radiotherapy was made, and treatment with colchicine and high-dose acetylsalicylic acid was initiated. Two weeks later, recurrent dyspnoea prompted readmission, and chest computed tomography excluded pulmonary embolism while confirming persistent pleural effusions. Corticosteroid therapy led to progressive clinical and radiographic improvement.

Follow-up Holter monitoring demonstrated a marked reduction in PVC burden to 6%, with recovery of effective biventricular pacing above 90%. No further arrhythmic symptoms or recurrent pericardial complications were observed. At six months of follow-up, the patient remained clinically stable under optimized heart failure therapy, with no additional cardiac or extracardiac radiation-related adverse effects.

This case illustrates the feasibility of ECGi-guided STAR as a noninvasive therapeutic option for refractory epicardial PVC impairing CRT efficacy. The integration of noninvasive electrical mapping with multimodality cardiac imaging enabled precise target definition and effective treatment delivery, highlighting the potential role of this approach in selected patients when conventional ablation strategies fail.

## Data Availability

No datasets were generated or analysed during the current study.
